# An open-source software ecosystem for the interactive exploration of ultrafast electron scattering data

**DOI:** 10.1186/s40679-018-0060-y

**Published:** 2018-09-22

**Authors:** Laurent P. René de Cotret, Martin R. Otto, Mark J. Stern, Bradley J. Siwick

**Affiliations:** 1 Department of Physics, McGill University, Montréal, Canada; 2 Department of Chemistry, McGill University, Montréal, Canada

**Keywords:** Ultrafast electron scattering, Visualization, Data processing, Open-source

## Abstract

This paper details a software ecosystem comprising three free and open-source Python packages for processing raw ultrafast electron scattering (UES) data and interactively exploring the processed data. The first package, *iris*, is graphical user-interface program and library for interactive exploration of UES data. Under the hood, *iris* makes use of *npstreams*, an extensions of *numpy* to streaming array-processing, for high-throughput parallel data reduction. Finally, we present *scikit-ued*, a library of reusable routines and data structures for analysis of UES data, including specialized image processing algorithms, simulation routines, and crystal structure manipulation operations. In this paper, some of the features or all three packages are highlighted, such as parallel data reduction, image registration, interactive exploration. The packages are fully tested and documented and are released under permissive licenses.

## Background

The advent of ultrafast electron diffraction (UES) has extended crystallography into the temporal dimension. Combining the spatial resolution of electron microscopy and femtosecond time-resolution, this laboratory-scale technique has shed light on a broad spectrum of phenomena, from photoinduced structural phase transitions in inorganic [21, 32] and organic materials [8], to coherent nuclear motion in dilute molecular gas [36]. Beyond probing structure through elastic interactions, UES has provided a direct observation of electron–phonon couplings and phonon relaxation pathways in layered materials [26, 31].

**Fig. 1:**
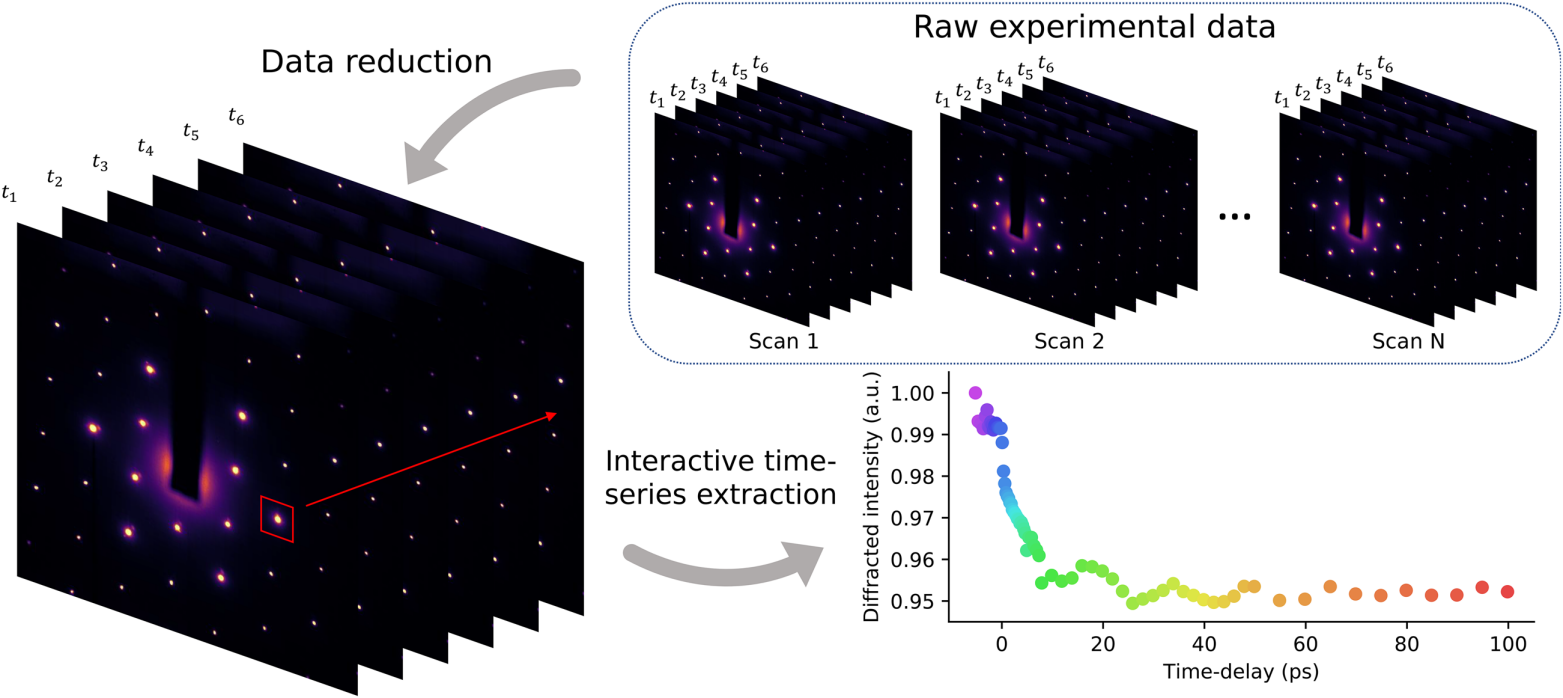
Ultrafast electron scattering data exploration workflow. To maximize signals, ultrafast electron scattering experiments consist in many identical sub-experiments (or scans), as shown on the top right. Data reduction consists in combining these sub-experiments, which usually involve image-alignment, intensity normalization, and other corrections. Each individual time-delay can be reduced in parallel. The resulting reduced data are a stack of scattering patterns, similar to a video. Scattering intensity time-series can be extracted from either a single pixel or integrated over a groups of pixels (lower right)

Ultrafast electron scattering is a stroboscopic technique. Electron scattering patterns are acquired a fixed time-delay after photoexcitation with an ultrafast laser. A full view of the scattering dynamics can be assembled by acquiring electron scattering patterns for sufficient time-delays. To maximize the signal-to-noise ratio, equivalent experiments—hereafter referred to as *scans*—are acquired sequentially. This set of equivalent sub-experiments forms the raw experimental data. In order to extract time-dynamics, the data must be distilled, or *reduced*; the data reduction might involve scattering intensity normalization or alignment, and ends in averaging. A physical picture of dynamics in a sample is built by looking at in scattering intensity over time, in a fixed region of reciprocal space. A conceptual view of the flow from raw data to dynamics is presented in Fig. 1.

The path from data acquisition to scientific insights can never be fully standardized and streamlined. However, one step common to all workflows is data exploration. This is especially important for time-resolved techniques such as UES due to the breadth and depth of information contained in a dataset. This emerging field would benefit greatly from an aggregation of efforts towards free and open-source tools that standardize and simplify analysis and interpretation of complex dynamics. In this work, we present a program built specifically for interactive data exploration, *iris*. We also introduce a software package of reusable routines and data structures related to ultrafast electron scattering (*scikit-ued*) and a streaming array operations package (*npstreams*), establishing a software foundation on which the community can build. *Iris* is the first integrated solution for interacting with UES data.

## Methods

The software ecosystem presented in this work is written in Python; the language is accessible to non-programmers, with a simple syntax and dynamic nature. The scientific Python community’s dedication to good documentation and focus on free and open-source packages are attractive features that played heavily in the decision to write iris in Python. While pure Python code typically results in poor performance, bottlenecks can easily be rewritten in a more efficient, compiled language, and seamlessly integrated with existing Python code [5]. It should be stressed that performance-critical parts of our software ecosystem are, in fact, thin layers of Python code on top of fast C libraries. The Python scientific stack is built on the *de facto* standard *numpy* package [29] that exports array operations rooted in compiled code, bypassing the slow nature of Python in most situations. Therefore, Python provides an ideal mix of performance and ease of development.

## Results and discussion

### Interactive data exploration

The primary tool presented in this work is *iris*, first and foremost a graphical user-interface (GUI) program that allows for interactive exploration of ultrafast electron scattering data. From its GUI, users can interact with raw data, process this raw data into a reduced dataset, enabling interactive data exploration. *Iris*’ second role consists of a library of data structures for handling ultrafast scattering data in external Python scripts and programs. Everything that can be done in the GUI can also be done programmatically.

The first step in the typical *iris* workflow involves interacting with raw scattering patterns from a variety of possible unique formats. For this purpose, *Iris* supports plug-ins that act as bridges between arbitrary data formats for raw data and *iris*’s internal representation.

**Fig. 2:**
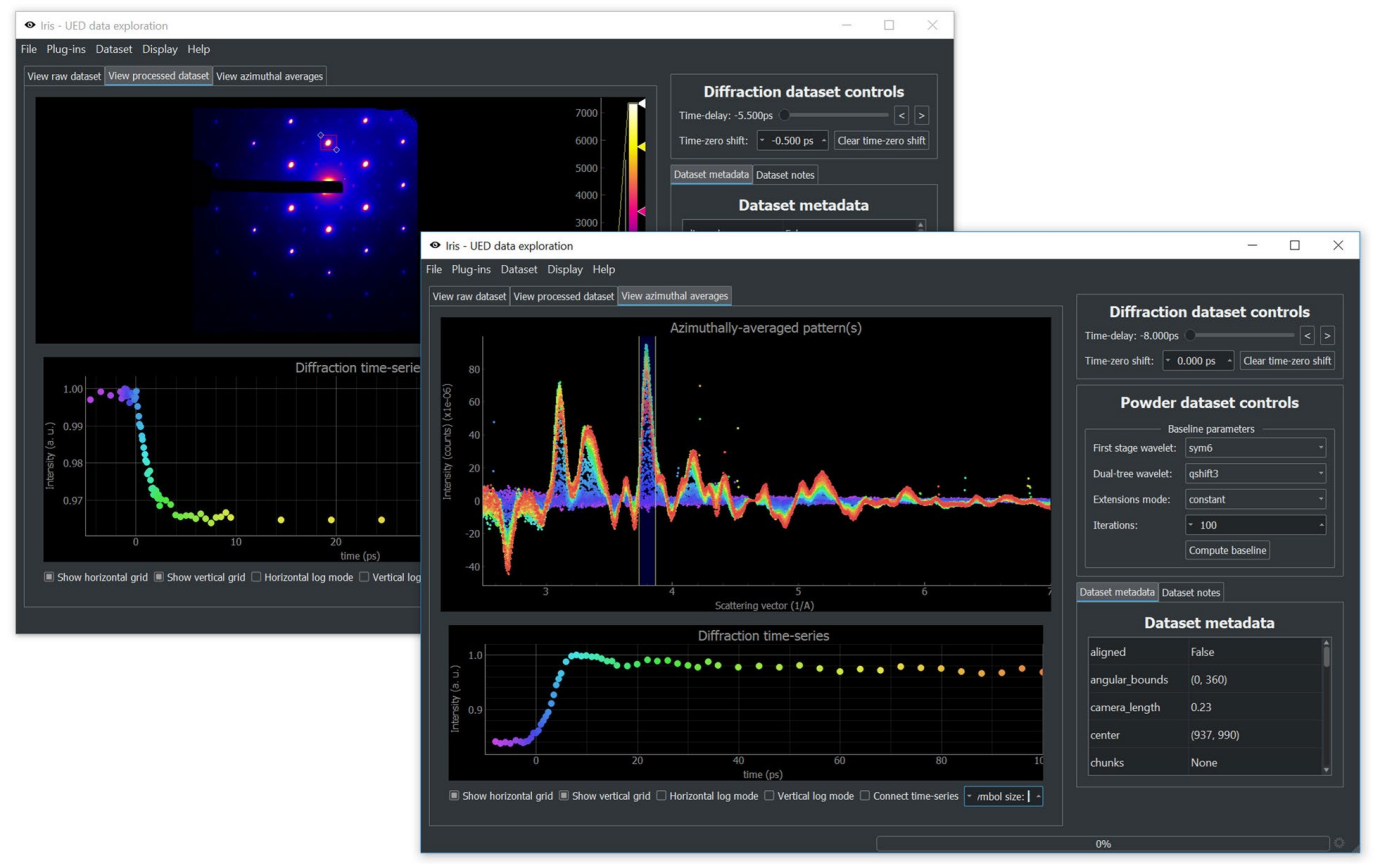
Overview of the GUI component of *iris*. Two GUI instances show typical datasets. On the top left, Bragg peak dynamics for photoexcited single-crystal data is shown. Diffracted intensity is integrated in the red square and its time dependence is shown in the bottom panel. On the bottom right, azimuthally averaged baseline-corrected polycrystalline scattering data are presented. The pre-photoexcitation scattering patterns have been subtracted so that dynamics are more evident. Diffraction patterns are color-coded based on their time-delay, shown below. Diffracted intensity is integrated inside the blue zone and its time dependence is again shown on the bottom panel. Both integration regions can be interactively dragged, updating the time-series in real-time

The second step in the typical *iris* workflow consists of data reduction. During this phase, equivalent scattering patterns acquired with the same time-delay are reduced in parallel. This reduction operation can involve image-alignment, intensity normalization, or any operation specified by a plug-in. Scattering patterns are then concatenated into a three-dimensional array, similar to a video. Experimental metadata, such as sample temperature, photoexcitation conditions, electron energy, and more, are also preserved. Arbirary metadata can be defined by users through the use of plug-ins. The performance optimization of the data reduction pipeline is discussed in depth in “Streaming operations on arrays” section.

*Iris* handles large reduced datasets by storing them using the Hierarchical Data Format version 5 (HDF5), an archival format designed for large *n*-dimensional numerical datasets [3, 13]. HDF5 supports transparent compression and data-corruption detection mechanisms. Most importantly, HDF5 supports data slicing, which allows to read specific portions of an HDF5 file without having to load the entire file—which can require tens of gigabytes of memory in the case of fully reduced UES datasets.

The final stage of the exploration workflow—time-series extraction—is specific to time-resolved techniques. Thanks to HDF5’s data slicing feature and careful performance optimizations within *iris*, dynamics in scattering patterns can be explored interactively, in real-time. Further data transformations are also possible, most importantly azimuthal averaging of scattering patterns from polycrystalline samples. Time-series extraction in the GUI is shown in Fig. 2 for two types of samples, single-crystal and polycrystal.

*Iris* was designed with compatibility in mind. For input compatibility, *iris*’s plug-in architecture allows to interface with arbitrary data formats. Writing a plug-in requires writing some Python code: users can then use the full power of the scientific Python stack to introduce extra processing in *iris*’s data reduction pipeline. In terms of output compatibility, *iris* datasets can be inspected and manipulated by a large variety of programs, thanks to HDF5’s official and unofficial bindings to programming languages such as C/C++, Fortran77/Fortran90, LabVIEW, MATLAB, Mathematica, R, Julia, Python, and many more. The HDF5 layout is described in the online documentation.

### Streaming operations on arrays

Raw scattering datasets can reach sizes up to hundreds of gigabytes. The reduction operation of concatenating images into dense *numpy* arrays, and then reducing—which usually involves scattering pattern alignment, intensity normalization, and finally a weighted average—is a slow process.

To effectively reduce the raw data, the package *npstreams* was created. *Npstreams* extends the core components of the array-oriented *numpy* package (called universal functions) to work on streams of arrays rather than dense, in-memory arrays. *Npstreams* can *automagically* generate streaming functions from *numpy* universal functions. The streams of arrays can be generated on-demand (such as images being progressively loaded from disk). Stream operations require much less memory, which in turns allows for parallelization. In fact, in the special case of stream operations on arrays of the same size (e.g., on images), data reduction can operate in constant-memory.

In the case of data reduction in *iris*, a stream of arrays is formed by progressively loading scattering patterns at same time-delay (but from different scans). Therefore, the reduction of a dataset of many time-delays and many scans to a dataset of many time-delays and a single scan can be done in parallel. In the limiting case where data processing performance is not bound by data loading time, the performance increase due to *npstreams* alone is *linear* in the number of processing units. Thus, an 8-core computer would experience an 8x performance increase by using *npstreams* in parallel.

**Fig. 3:**
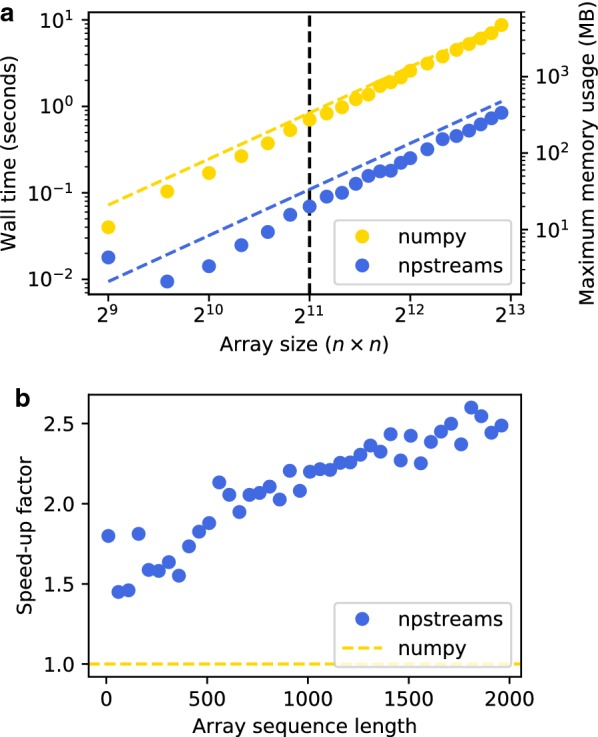
Performance characterization of the *npstreams* package in comparison with *numpy*. The task was to average sequences of 2D arrays (representing scattering patterns). **a** Wall time of averaging for a sequence of 10 arrays (solid) superimposed with maximum memory usage (dashed). The vertical-dashed line marks the array size of $2048 \times 2048$, equivalent to a scattering pattern of 4 megapixels. **b** Speed-up of using *npstreams* vs. *numpy* for averaging a sequence of arrays of size $512 \times 512$ elements

Single-core performance is also improved. To measure single-core performance, benchmarks set up a sequence of $n \times n$ arrays of random floating-point numbers representing scattering patterns. Then, arrays are either directly averaged using *npstreams*, or concatenated into a dense ndarray which is in turns averaged using *numpy*. The results of running this benchmark on sequence of varying lengths and arrays of varying sizes is presented in Fig. 3.

While the core of *npstreams* is concerned with autogenerating streaming functions from basic universal *numpy* functions, some more complex streaming functions are implemented, with the aim of providing real-world examples. For instance, a streaming weighted average and streaming weighted standard deviation routines are included, based on West [33].

**Fig. 4:**
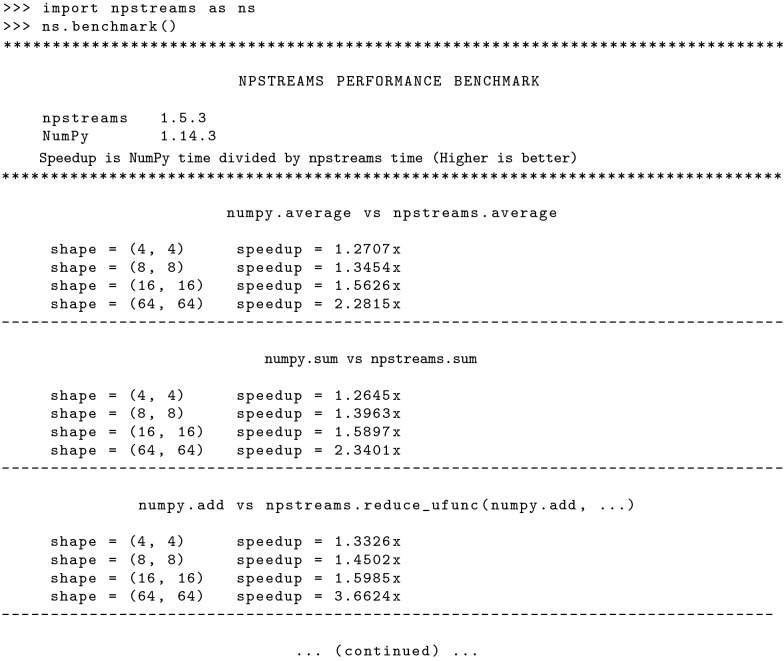
How to run *npstreams* benchmarks suite from the interactive Python interpreter. By default, a pre-selected set of functions from both the *numpy* and *npstreams* packages are compared

*Npstreams* also includes benchmarking functionality, where users can generate benchmark results on their machine with a simple command, as shown in Fig. 4.

The functionality of *npstreams* extends beyond the requirements of *iris* and *scikit-ued*. Streaming array operations can deal with an infinite stream of arrays. For example, an operation can be applied until certain criteria are met, or a pipeline can be assembled which can run forever, in constant (low) memory.

### Reusable routines and data structures for ultrafast electron scattering data analysis

The scientific Python community has access to over 50 research-oriented packages called *scikits*, extensions of the general-purpose SciPy package [17]. These software packages provide routines and algorithms for handling image processing (*scikit-image* [30]), performing machine-learning tasks (*scikit-learn* [24]), interacting with spectroscopy data (*scikit-spectra*), bio-informatics (*scikit-bio*), and much more. In the tradition of these scikits, we introduce *scikit-ued*, a collection of algorithms, routines, and data structures commonly needed for ultrafast electron scattering data interactions. Most operations *iris* performs on datasets are offloaded to *scikit-ued*.

*Scikit-ued* provides a repository of reusable components in many domains, such as image analysis, crystal structure manipulation, baseline-removal, simulation, common utilities, and more. Some of its features are described in the following sections.

#### Baseline-removal

**Fig. 5:**
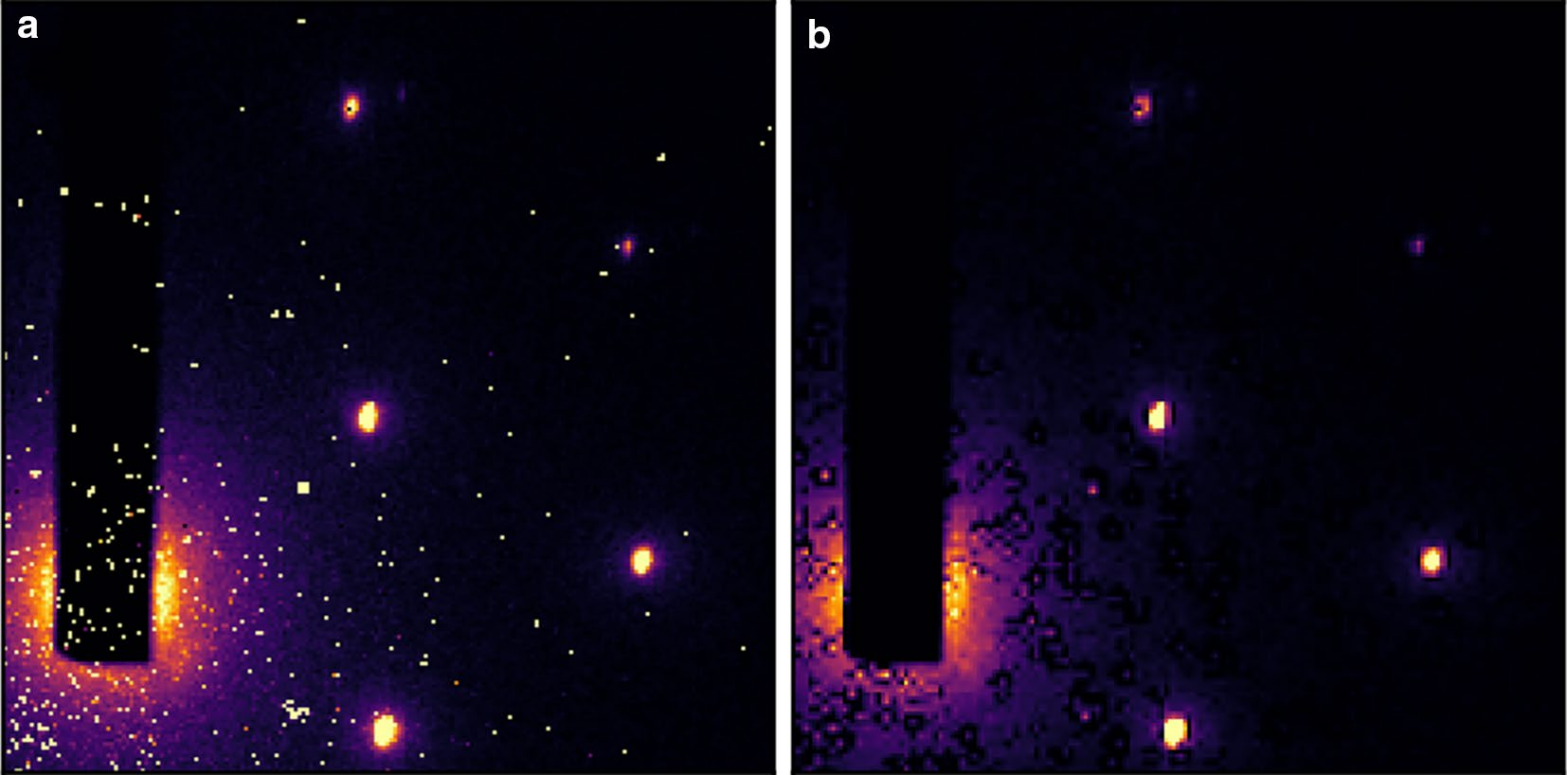
Removing image hotspots using an iterative baseline-removal algorithm based on the discrete wavelet transform. **a** Original scattering pattern shows hotspots due to laser light hitting the detector. **b** Baseline of **a**. The hotspots are treated as the foreground by the iterative algorithm

The baseline-removal functionality provided by *scikit-ued* is the evolution of the approach previously published by the authors. Readers interested in the details are referred to [6]. Some extensions have been made, such as the ability to compute a background for 2D images using the iterative algorithm based on the discrete wavelet transform presented in [7]. This approach can be used to remove blemishes on images: by treating hot spots or defects as foreground, the baseline of the original image will show no hotspots. An example of this is shown in Fig. 5.

#### Image analysis

Scattering patterns analysis intersects with general image processing in many ways. In most cases, other libraries such as *scikit-image* can be used. Specific tasks not widely available elsewhere have been included in *scikit-ued*.

**Fig. 6:**
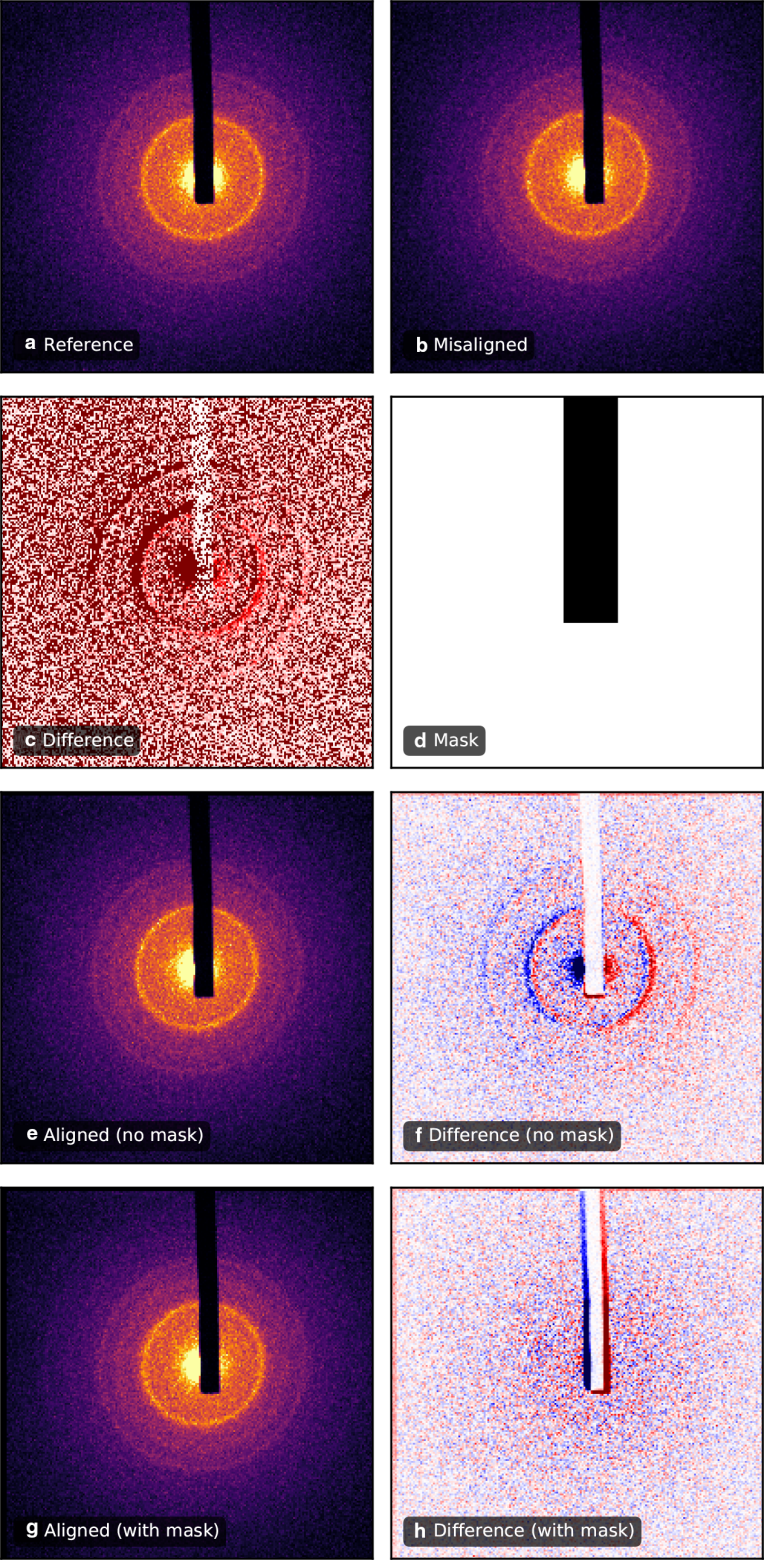
Scattering pattern alignment based on the masked normalized cross-correlation algorithm. **a** Reference scattering patterns of polycrystalline chromium. **b** Misaligned scattering pattern. **c** Difference between the patterns in **a** and **b** shows structure indicative of a sideways shift. Note that the beam block has not moved. **d** Pixel mask of the beam block. Black pixels represent zones to be ignored by the alignment procedure. **e** Aligned image, registered without the mask in **d**. **f** Difference between **a** and **e** shows residual misalignment. **g** Aligned image, registered with the mask in **d**. **f** Difference between **a** and **g** shows successful alignment

A common data processing task, implemented in the data reduction pipeline of *iris*, is scattering pattern alignment to a reference. During the course of data acquisition, scattering patterns can drift across the detector due to fluctuations in the electron beam alignment, while other image components (e.g., beam-stop, hard edges, detector defects) will appear static. Therefore, cross-correlation techniques used in image registration generally fail at scattering pattern alignment. *Scikit-ued* includes an advanced image-alignment algorithm, known as the masked normalized cross-correlation image registration [22]. By masking static components of scattering data, the image registration algorithm will only compare misaligned sections, greatly enhancing registration accuracy. An example of this algorithm is presented in Fig. 6.

**Fig. 7:**
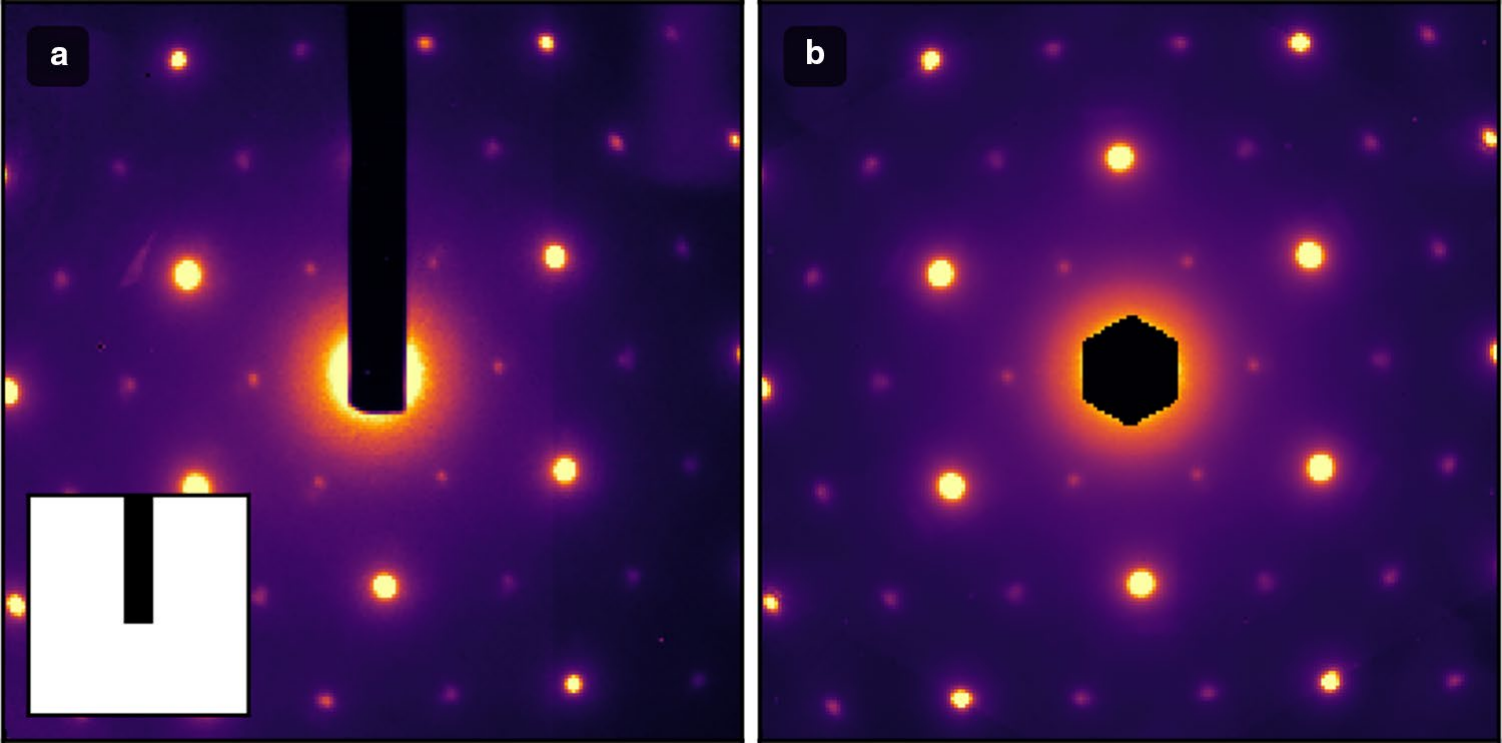
Example of symmetrization of single-crystal scattering patterns. **a** Raw single-crystal scattering pattern. Inset shows the pixel mask indicating where the beam-block is located. Pixels under the mask are ignored during the symmetrization routine. **c** Sixfold rotational symmetrization of scattering pattern in **a** with beam-block masked reduces noise and enhances dynamics

Symmetry-based operations are also implemented in *scikit-ued*. One such operation is azimuthal averaging, a procedure during which polycrystalline scattering patterns are reduced to one radial dimension. *Scikit-ued* includes discrete symmetry-based operations as well, most importantly discrete rotational averaging. Scattering patterns exhibiting *n*-fold rotational symmetry can be transformed to yield a higher signal-to-noise ratio. This approach has recently been used to extract small ultrafast diffuse scattering signals from graphite [26]. An example of such symmetrization is presented in Fig. 7.

#### Structure manipulation

**Fig. 8:**
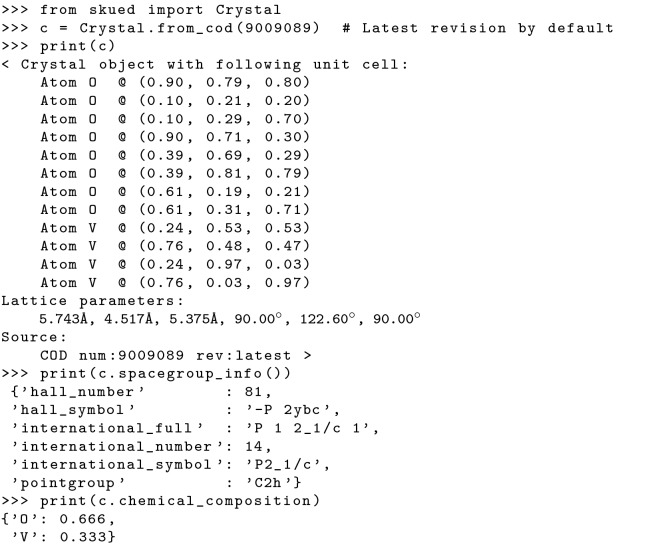
Generating a Crystal instance for vanadium dioxide from the Crystallography Open Database entry 9009089 [34], inside the interactive Python interpreter

*Scikit-ued* includes data structures that make it easy to read and manipulate crystallographic information. The Crystal class is the primary data structure giving access to atomic positions, chemical composition, lattice information, and more. It can be generated from a few types of sources, with the most convenient type involving the ubiquitous Crystal Information File (CIF) format [2, 15]. Structures can also be pulled from entries in the Crystallography Open Database [9, 10], as well as generated from Protein Data Bank entries [1, 14]. A number of structures (mostly simple elemental crystals) are included with the package. Finally, Crystal instances can be generated from (and converted to) the Atomic Simulation Environment Atoms format [20]. An example of structure manipulation is presented in Fig. 8.

Once a Crystal instance has been created, space-group information and symmetry operations can be determined through the library spglib [12, 27]. Using this information, crystals can also be reduced to their primitive cells.

#### Simulation

**Fig. 9:**
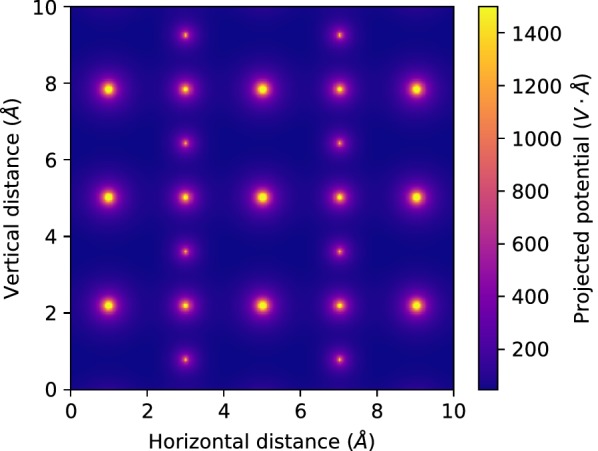
Simulated electrostatic potential of orthorhombic barium titanate (BaTiO$_3$) projected onto the $z=0$ plane Structure file was taken from [35]

*Scikit-ued* has built-in support for calculation of crystal potentials, based on the parametrization of Kirkland [18]. The example of real-space, projected electrostatic potential of orthorhombic barium titanate (BaTiO$_3$) is shown in Fig. 9. These calculations are required in the implementation of multislice simulations [4, 19].

**Fig. 10:**
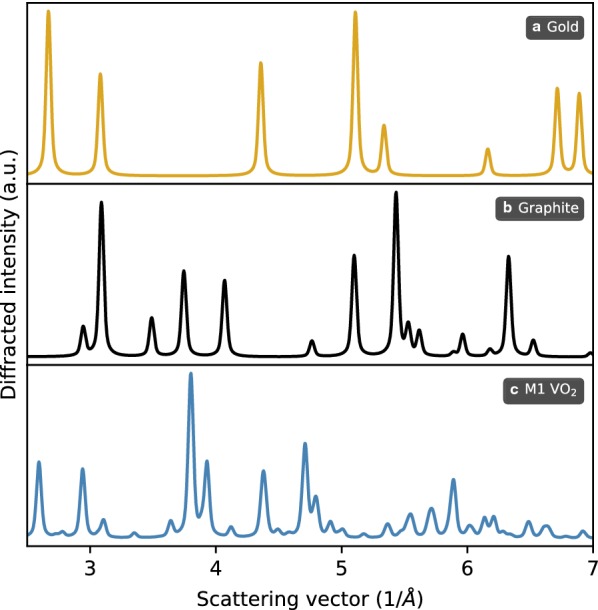
Simulated polycrystalline electron diffraction patterns for gold, graphite, and monoclinic M1 VO$_2$

From the parametrization of atomic potentials, the atomic form factors can also be calculated—and from them static structure factors. *Scikit-ued* exports a routine for simulating electron diffraction patterns for polycrystalline samples based on the atomic positions of Crystal objects. Examples of simulated diffraction patterns from built-in *scikit-ued* structures are presented in Fig. 10.

#### Input and output

*Scikit-ued* provides routines to read exotic image file formats. *Scikit-ued* can read Merlin Image Binary files (*.mib)—both single image and multi-images files—as well as Gatan’s proprietary DM3 and DM4 formats (*.dm3, *.dm4). *Scikit-ued* can also read all images supported by *scikit-image*, notably images encoded in the Tagged Image File Format (*.tiff).

#### Miscellaneous

Many small utilities are included in *scikit-ued*. Fast computation of pseudo-voigt profiles is included and integrated with the polycrystalline diffraction simulation routine, based on the work by Ida et al. [16]. Calculation of thin film optical properties is also implemented, based on Tomlin [28]. Finally, a preliminary version of the non-uniform Fast Fourier Transform (NFFT) is available [11]. Additional functionality is presented in the documentation.

### Common features

All three libraries presented herein benefit from some common features and development tools.

First and foremost, all three packages are documented online (offline documentation is also available). Reference documentation is automatically generated from the source code, which limits the possibility of documentation being out-of-date with respect to the source code [23]. The documentation for all three packages also includes hand-written tutorials. The documentation for each package is hosted online by Read the Docs and links are specified in see “Availability of data and materials” section.

The repositories are hosted on GitHub, a web-based code hosting service with built-in version control through Git. As the packages are free and open-source, GitHub can be used to browse source code. It also provides features not available through Git itself, most importantly an issue tracker. This issue tracker is also directly accessible from the *iris* GUI through a help menu. Bugs and issues raised through GitHub are publicly visible and provide a place to discuss potential solutions.

After changes are committed to one of the repositories, the updated package is automatically installed and tested in a remote environment—a practice known as continuous integration. Committed changes also trigger the automatic generation of a new documentation version, which is then posted online within minutes.

Finally, stable versions of all three packages are uploaded to the Python Packaging Index (PyPI), where they can be inspected and downloaded. For users of the Anaconda Python distribution, the three packages are also available for install within the conda environment, which provides pre-compiled packages.

### Roadmap

The natural progression for *iris* involves data exploration along different dimensions beyond time: diffracted intensity along dimensions of fluence, temperature, doping concentration, and more.

The target functionality of *npstreams* has largely been implemented. The obvious key improvement concerns performance, which could be increased by rewriting the core functionality in C, while exploiting *numpy*’s C interface.

Future developments of *scikit-ued* concern the simulation subpackage. Simulation of polycrystalline scattering pattern is indispensable as a tool to test methods and validate hypotheses; simulation of single-crystal scattering patterns is a natural evolution of *scikit-ued*’s capabilities. While the basic parametrizations of atomic properties are already implemented, as well as the computation of real-space electrostatic potential of arbitrary crystal structures (see Fig. 9), wave-propagation calculations—e.g., the multislice algorithm—remain to be implemented. See [25] for a summary of available electron scattering and microscopy simulation tools to which *scikit-ued* could bind.

## Conclusion

An ecosystem of three free and open-source Python packages for exploring ultrafast electron scattering data was presented. This ecosystem, governed by permissive licenses, was created with collaboration in mind, while adhering to sane software development practice. We introduced *npstreams*, a streaming array-processing library that allows for constant-memory data reduction. *Scikit-ued*, a *scipy* extension that contains algorithms and data structures for the analysis of UES data, was also presented. Finally, the ecosystem culminates in the *iris* package, the first integrated data exploration interface specifically tailored to the data-rich nature of UES. *Iris*’ plug-in functionality as well as real-time, interactive capabilities pushes the limits of what is possible in field of ultrafast electron scattering.

## Abbreviations

UES: ultrafast electron scattering
GUI: graphical user-interface
HDF5: hierarchical data format version 5
CIF: crystal information file
COD: crystallography open database
PyPI: Python packaging index
